# Identification and characterization of a vitamin D_3_ decomposition product bactericidal against *Helicobacter pylori*

**DOI:** 10.1038/srep08860

**Published:** 2015-03-09

**Authors:** Kouichi Hosoda, Hirofumi Shimomura, Kiyofumi Wanibuchi, Hisashi Masui, Avarzed Amgalanbaatar, Shunji Hayashi, Takashi Takahashi, Yoshikazu Hirai

**Affiliations:** 1 Division of Bacteriology, Department of Infection and Immunity, Jichi Medical University, 3311-1, Yakushiji, Shimotsuke-shi, Tochigi. 329-0498, Japan; 2 Faculty of Pharmaceutical Sciences, Yokohama College of Pharmacy, 601, Matano-cho, Totsuka-ku, Yokohama, Kanagawa. 245-0066, Japan; 3 Department of Microbiology, Kitasato University School of Medicine, 1-15-1, Kitasato, Minami-ku, Sagamihara, Kanagawa. 252-0374, Japan

## Abstract

This study demonstrated that the vitamin D_3_ decomposition product VDP1 exerts an antibacterial action against *Helicobacter pylori* but not against other bacteria. Treatment with VDP1 induced a collapse of cell membrane structures of *H. pylori* and ultimately lysed the bacterial cells. A unique dimyristoyl phosphatidylethanolamine in the membrane lipid compositions contributed to the interaction of VDP1 with *H. pylori* cells. In separate experiments, VDP1 had no influence on the viability of the human cancer cell lines MKN45 and T47D and lacked any vitamin D_3_-like hormonal action against the latter. In both ^1^H and ^13^C NMR analyses, the spectra patterns of VDP1 corresponded with those of Grundmann's ketone. These results suggest that VDP1 (or Grundmann's ketone-type indene compound) may become a fundamental structure for the development of new antibacterial substances with selective bactericidal action against *H. pylori*.

H*elicobacter pylori* colonizes the human gastric epithelium in approximately half of the world's population. The bacterium takes the form of a curved Gram-negative rod equipped with flagella for motility, and can only grow in microaerophilic environments^1^. As a pathogen, *H. pylori* causes chronic gastritis and peptic ulcers in infected individuals, and persons who carry the bacteria for many years face an elevated risk of gastric cancer and gastric mucosa-associated lymphoid tissue lymphoma^2^,^3^. *H. pylori*-infected patients are medicated with antibacterial substances such as amoxicillin, clarithromycin, or metronidazole. Year by year, however, *H. pylori* strains resistant to these medicines have been increasing^4^,^5^. If the outbreak of resistant strains is to be prevented, it will be essential to develop new antibacterial agents that act selectively on unique biological features of *H. pylori*. It will also be essential, in developing new anti-*H. pylori* agents, to ensure that the agents confer no effects on the survival or drug-resistant expression of other bacterial species, especially human intestinal bacteria.

Recent studies by our group have revealed that phosphatidylethanolamine (PE) in *H. pylori* lipid compositions carries a myristic acid molecule, has a high binding affinity to non-esterified steroidal compounds such as free cholesterol, pregnenolone, and progesterone at the carbon-3 position in its frames, and plays an important role in the interaction between the bacterial cells and these steroidal compounds^6^,^7^. We have also found that dimyristoyl PE (DMPE) is one of the most prevalent PE molecular species in *H. pylori* cell membrane lipid compositions. Of the steroidal compounds, progesterone and its analogues induce the cell lysis of *H. pylori* by binding to the myristoyl PE molecular species of the bacterial cells^7^,^8^. These investigations by our group suggest that chemical compounds with high binding affinity to the *H. pylori* PE may confer selective antibacterial action against this bacterium.

Vitamin D_3_ is a steroidal compound in which the carbon-3 position in the seco-steroid frame is non-esterified with a side chain such as a fatty acid molecule. The carbon position-3 of two vitamin D_3_ metabolites, namely, the circulating form of vitamin D_3_ (25-hydroxyvitamin D_3_) and activated form of vitamin D_3_ (1α,25-dihydroxyvitamin D_3_), also lacks an acyl group in the seco-steroid frame. No earlier investigations, however, have elucidated the interaction of the three vitamin D_3_ species with *H. pylori* cells. We therefore analyzed the interaction of vitamin D_3_ species with *H. pylori* cells.

## Results

### Antibacterial activities of three vitamin D_3_ species against *H. pylori*

**Figure 1:**
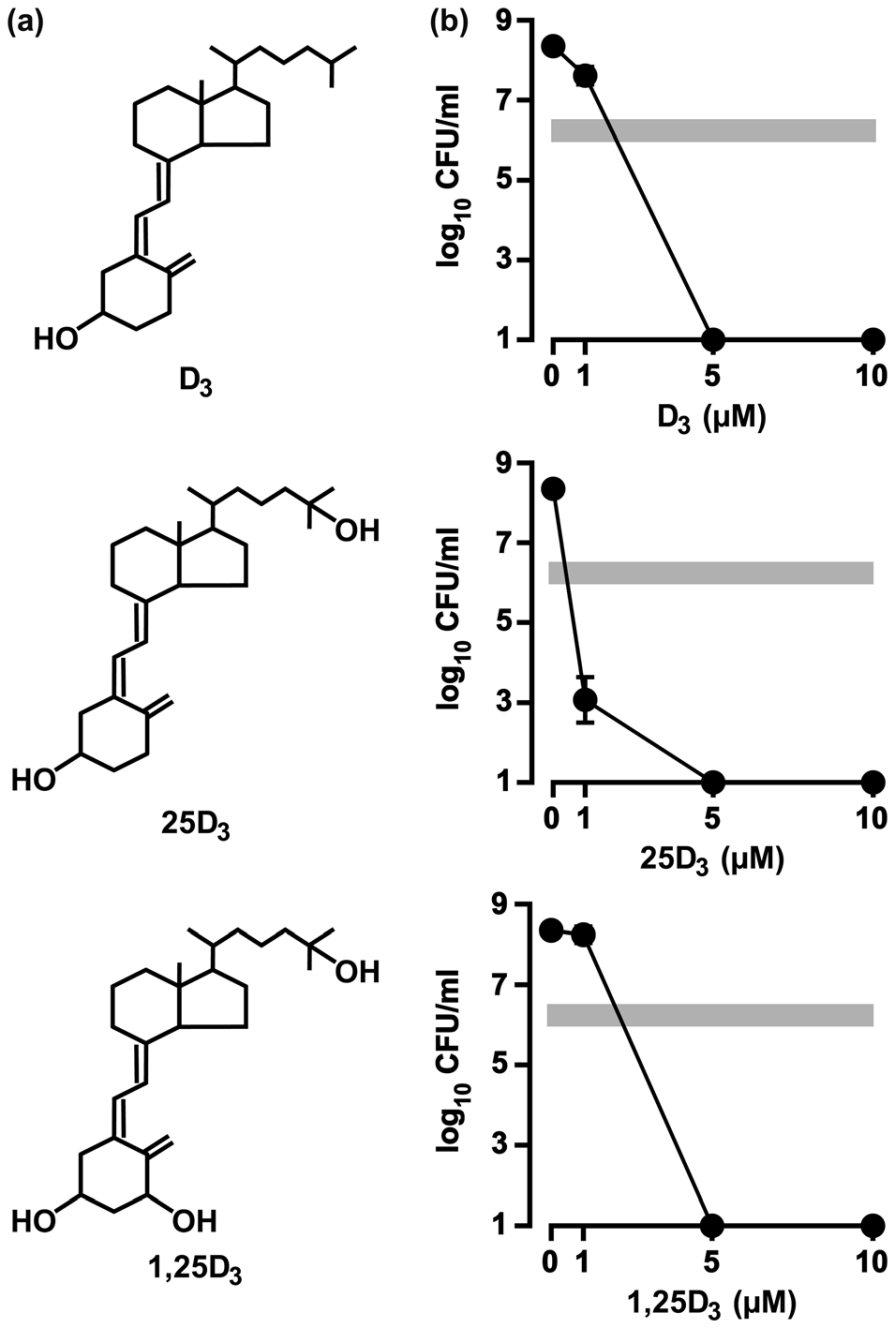
Antibacterial activities of the three vitamin D_3_ species against *H. pylori*. (a) The chemical structures of vitamin D_3_ (D_3_), 25-hydroxyvitamin D_3_ (25D_3_), and 1α,25-dihydoxyvitamin D_3_ (1,25D_3_). (b) FC-free *H. pylori* strain NCTC 11638 was incubated for 24 h in the presence of each D_3_ species in a broth, and the CFUs were counted. The gray bars in the graphs represent the baseline CFU count measured immediately after the incubation was started. Three independent experiments with the respective D_3_ species were performed to calculate the mean CFU ± SD per ml.

When *H. pylori* without free cholesterol (FC) was incubated for 24 h in the presence of the vitamin D_3_ species at the concentrations ranging from 1 to 10 μM, every vitamin D_3_ species at the 5 μM concentration suppressed the CFU count to below the limits of detection (Fig. 1b). In sum, all three vitamin D_3_ species tested exhibited the antibacterial action against *H. pylori*.

### Alteration of the anti-*H. pylori* activities of the vitamin D_3_ species via non-biological degradations

**Figure 2:**
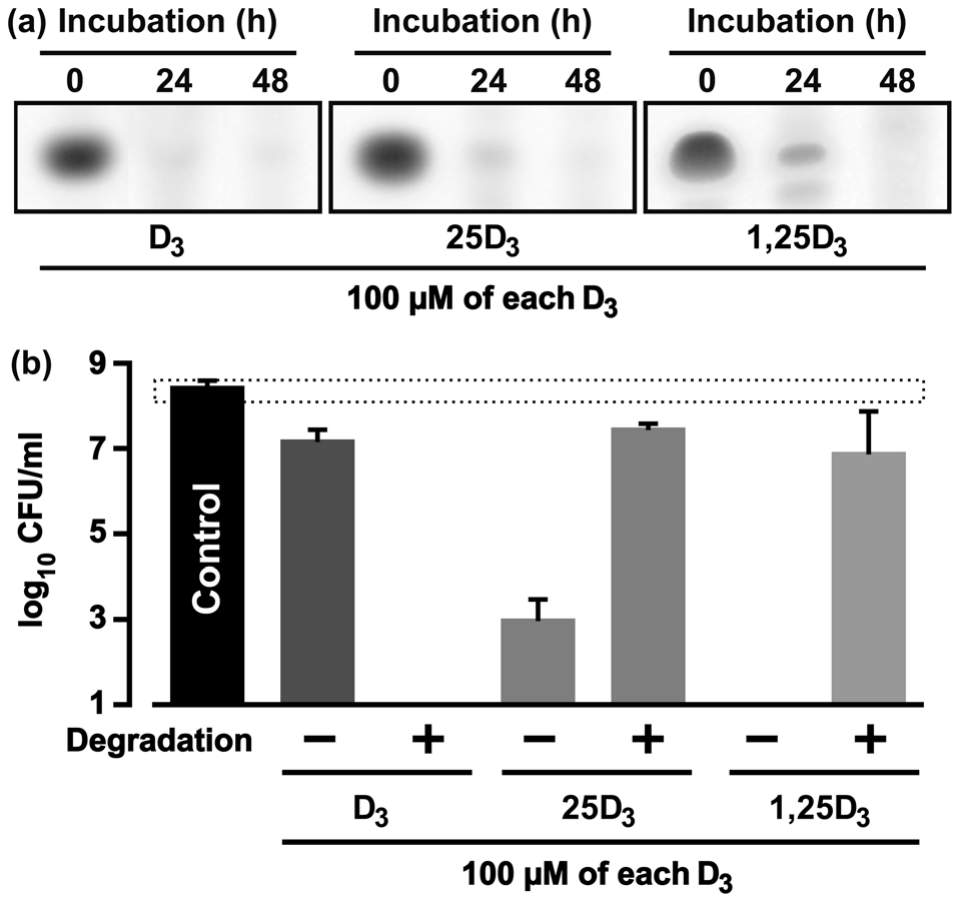
Antibacterial activities of decomposed vitamin D_3_ species against *H. pylori*. (a) Three vitamin D_3_ species dispersed into distilled water were incubated for 24 to 48 h at 70°C, recovered via the organic solvent distribution method, and analyzed by TLC. D_3_, vitamin D_3_; 25D_3_, 25-hydroxyvitamin D_3_; 1,25D_3_, 1α,25-dihydroxyvitamin D_3_. (b) The decomposition products obtained from the 48-h incubation of the experiments described in panel (a) were incubated for 2 h in the bacterial suspensions of FC-free *H. pylori*, and the CFUs were then counted. The minus and plus symbols in the graph are the CFU levels of *H. pylori* incubated in the presence of the intact D_3_ species and decomposed D_3_ species, respectively. The control in the graph is the CFU level of *H. pylori* incubated without any D_3_ specimens. The dashed line bar in the graph represents the baseline CFU measured immediately after the incubation was started. Three independent experiments with the respective D_3_ species were performed to calculate the mean CFU ± SD per ml.

Seco-steroids are known to be structurally unstable compounds. Vitamin D_3_ is biologically metabolized or degraded via the catalytic action of hydroxylases of cytochrome P450 families such as CYP11A1, CYP27A1, CYP27B1 and CYP24A1^9^. In addition, vitamin D group has been shown to non-biologically decompose via the exposure to high humidity and high temperature^10^. This study adopted the non-biological degradation of vitamin D_3_ species and investigated the antibacterial activity of the degraded vitamin D_3_ species against *H. pylori*. We dispersed the three vitamin D_3_ species for 24 to 48 h into distilled water warmed at 70°C in order to decompose the compounds and analyze the decomposition by TLC. The TLC analysis detected conspicuous reductions in the spot densities of all three vitamin D_3_ species examined in both 24-h and 48-h incubations (Fig. 2a). Each of vitamin D_3_ species decomposed via the 48-h incubation in distilled water (70°C) was added into a bacterial suspension of FC-free *H. pylori* and shaken for 2 h. The antibacterial actions of 25-hydroxyvitamin D_3_ and 1α,25-dihydroxyvitamin D_3_ against *H. pylori* were conspicuously attenuated by the heat degradation of the compounds. As a consequence, the CFU counts of *H. pylori* were reduced more in the presence of intact 25-hydroxyvitamin D_3_ or intact 1α,25-dihydroxyvitamin D_3_ than in the presence of decomposed 25-hydroxyvitamin D_3_ or decomposed 1α,25-dihydroxyvitamin D_3_ (Fig. 2b). On this basis, 25-hydroxyvitamin D_3_ and 1α,25-dihydroxyvitamin D_3_ were found to directly act on *H. pylori* cells and ultimately eradicate them. Unlike 25-hydroxyvitamin D_3_ and 1α,25-dihydroxyvitamin D_3_, the heat-degraded vitamin D_3_ conferred a remarkably augmented antibacterial action against *H. pylori*. Hence, the CFU counts of *H. pylori* were reduced more in the presence of decomposed vitamin D_3_ than in the presence of intact vitamin D_3_. These results indicate that the antibacterial action of vitamin D_3_ against *H. pylori* depended on a certain decomposition product resulting from the warming of the vitamin D_3_ dispersed into the distilled water.

### Antibacterial activities of vitamin D_3_ decomposition products against *H. pylori*

**Figure 3:**
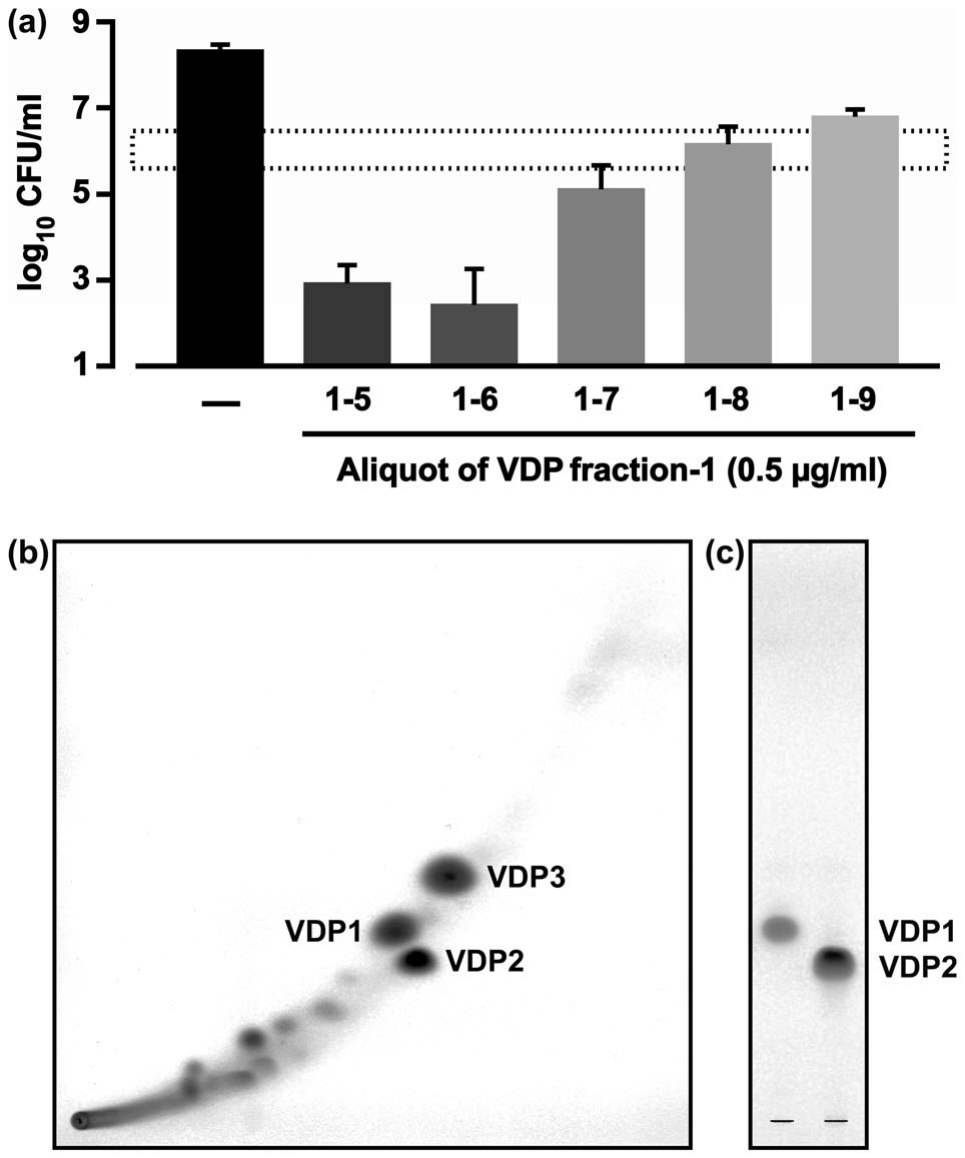
Anti-*H. pylori* activities in the aliquots of VDP fraction-1, and TLC analysis of VDP1 and VDP2. (a) FC-free *H. pylori* was incubated for 24 h in a broth containing aliquoted fraction-1 to measure the CFUs. The dashed line bar in the graph represents the baseline CFU count. Three independent experiments were performed to calculate the mean CFU ± SD per ml. (b) The collected fractions 1–5 and 1–6 (200 μg) were analyzed by 2-dimensional TLC. (c) The purified VDP1 (50 μg) and VDP2 (50 μg) were analyzed by TLC.

Next, we dispersed vitamin D_3_ for 1 week into distilled water warmed at 70°C to obtain vitamin D_3_ decomposition products (VDPs) in abundant amounts, divided the VDPs into four fractions by column chromatography, and investigated the anti-*H. pylori* activities in the divided fractions. When the FC-free *H. pylori* was incubated for 24 h in a broth containing a 0.5 μg/ml concentration of each VDP fraction, the most conspicuous decrease in CFU counts was observed in *H. pylori* incubated with VDP fraction-1. Next, we further divided the VDP fraction-1 into nine aliquots by column chromatography and investigated the anti-*H. pylori* activities in each of the aliquots. When FC-free *H. pylori* was incubated for 24 h in a broth containing a 0.5 μg/ml concentration of each aliquot, the most conspicuous reduction in CFU counts was observed in *H. pylori* incubated with aliquoted fraction 1–5 or 1–6 (Fig. 3a).

### Purification of VDP1 and VDP2 from the VDP fraction-1

We next performed a 2-dimensional TLC analysis of the aliquots collected from the VDP fractions 1–5 and 1–6. The TLC analysis detected three high-density spots of VDP (Fig. 3b). We then tried to purify the three VDPs via HPLC, and were successful in purifying two, VDP1 and VDP2 (Fig. 3c). VDP3, the third VDP, seemed to be structurally unstable or to volatilize easily, as it disappeared during the purification.

### Antibacterial activities of VDP1 and VDP2 against *H. pylori*

**Figure 4:**
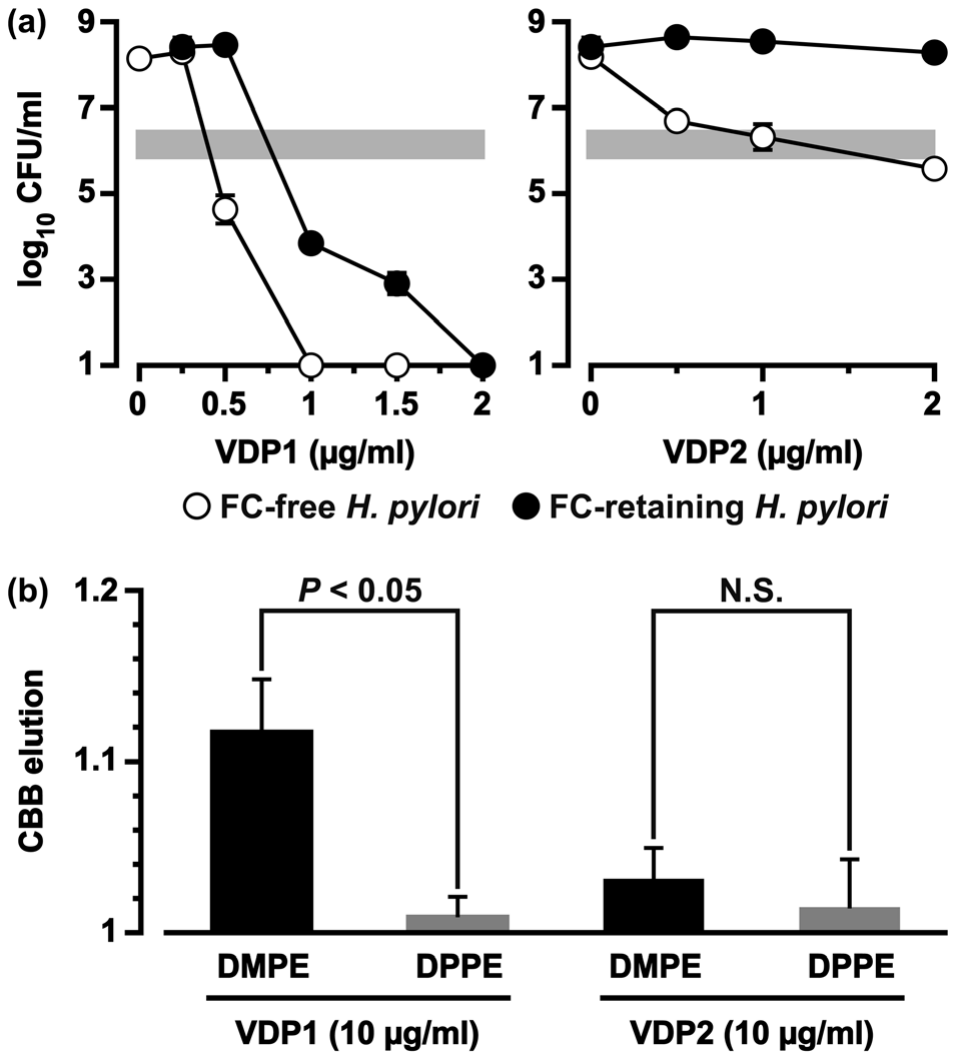
Anti-*H. pylori* action of the purified VDP1 and VDP2, and interaction of the VDPs with the PE vesicles. (a) FC-free *H. pylori* or FC-retaining *H. pylori* was incubated for 24 h in the presence of either VDP1 or VDP2 in a broth with or without FC (30 μM), and the CFUs were counted. The gray bars in the graphs represent the baseline CFU counts. The mean CFU ± SD per ml was obtained from three independent experiments. (b) PE vesicle suspensions containing CBB were shaken for 2 h in the presence of either VDP1 or VDP2, and A_590 nm_ was measured in the supernatant. CBB elution was denoted by the relative A_590 nm_ to the A_590 nm_ measured as a value of 1 in the supernatant of the PE vesicles incubated without VDPs. The statistical significance of differences between the CBB elutions from the DMPE vesicles and DPPE vesicles was evaluated by the *t*-test based on data obtained from three independent pair-experiments. N.S. stands for “no statistical significance”.

When FC-free *H. pylori* was incubated for 24 h in the presence of either VDP1 or VDP2 at concentrations ranging from 0.5 to 2 μg/ml, VDP1 at the 1 μg/ml concentration suppressed the CFU count of FC-free *H. pylori* to below the limits of detection (Fig. 4a). VDP2 exerted a weaker antibacterial action against the FC-free *H. pylori* than VDP1. The CFUs were thus maintained at the baseline level even in the presence of VDP2 at the highest concentration, 2 μg/ml. Earlier studies by our group have demonstrated that *H. pylori* acquires resistance to the antimicrobial actions of phosphatidylcholines, antibiotics, and bile salts via the incorporation of FC into the cell membranes^11^,^12^,^13^. To investigate further in the present study, we examined the antibacterial activities of VDP1 and VDP2 against FC-retaining *H. pylori*. When the same experiments performed on FC-free *H. pylori* were performed on FC-retaining *H. pylori* in a broth containing 30 μM FC, VDP1 at the 2 μg/ml concentration suppressed the CFU count to below the limit of detection (Fig. 4a). Meanwhile, the antibacterial effect of VDP2 against the FC-retaining *H. pylori* was completely abolished. Hence, the CFU count of this phenotypic *H. pylori* in the presence of VDP2 at the 2 μg/ml concentration was comparable to the CFU count in the absence of VDP2.

### Binding affinity of VDP1 and VDP2 to PE vesicles

Recent studies by our group have revealed that *H. pylori* PE carrying a myristic acid molecule plays an important role in the selective binding of non-esterified steroidal compounds at the carbon-3 position in its frame and that *H. pylori* cell membranes interact with those steroidal compounds via the mediation of the PE molecular species^6^,^7^. We next examined whether VDP1 and VDP2 derived from a steroidal compound (vitamin D_3_) have a higher binding affinity to PE molecular species carrying two myristic acid molecules (DMPE) than to PE molecular species carrying two palmitic acid molecules (DPPE). A suspension of PE vesicles containing Coomassie brilliant blue (CBB) prepared with either DMPE or DPPE was shaken for 2 h in the presence of the 10 μg/ml concentration of VDP1 or VDP2, and the absorbance of CBB eluted from the PE vesicles was measured in the supernatants at a wavelength of 590 nm. The level of CBB elution from DMPE vesicles incubated with VDP1 was statistically higher than the level of CBB elution from DPPE vesicles incubated with VDP1 (Fig. 4b). This tell us that DMPE vesicles interact more strongly with VDP1 than DPPE vesicles, and that VDP1 destabilizes the vesicle structure of DMPE and induces CBB elution from the vesicles. In contrast, the levels of CBB elution from the DMPE and DPPE vesicles incubated with VDP2 did not statistically differ. Hence, VDP2 has a low binding affinity to both DPPE vesicles and DMPE vesicles. Given that PE is the most predominant glycerophospholipid in the cell membrane lipid compositions of *H. pylori*^14^, and given that DMPE is one of the most prevalent PE molecular species of this bacterium^6^, we can assume that VDP1 at least binds to the PE carrying two myristic acid molecules on the *H. pylori* cells. Based on the results shown in Fig. 4, we paid attention to VDP1 and performed the detailed experiments for clarifying the anti-*H. pylori* activity of VDP1.

### Bactericidal mechanism of VDP1 against *H. pylori*

**Figure 5:**
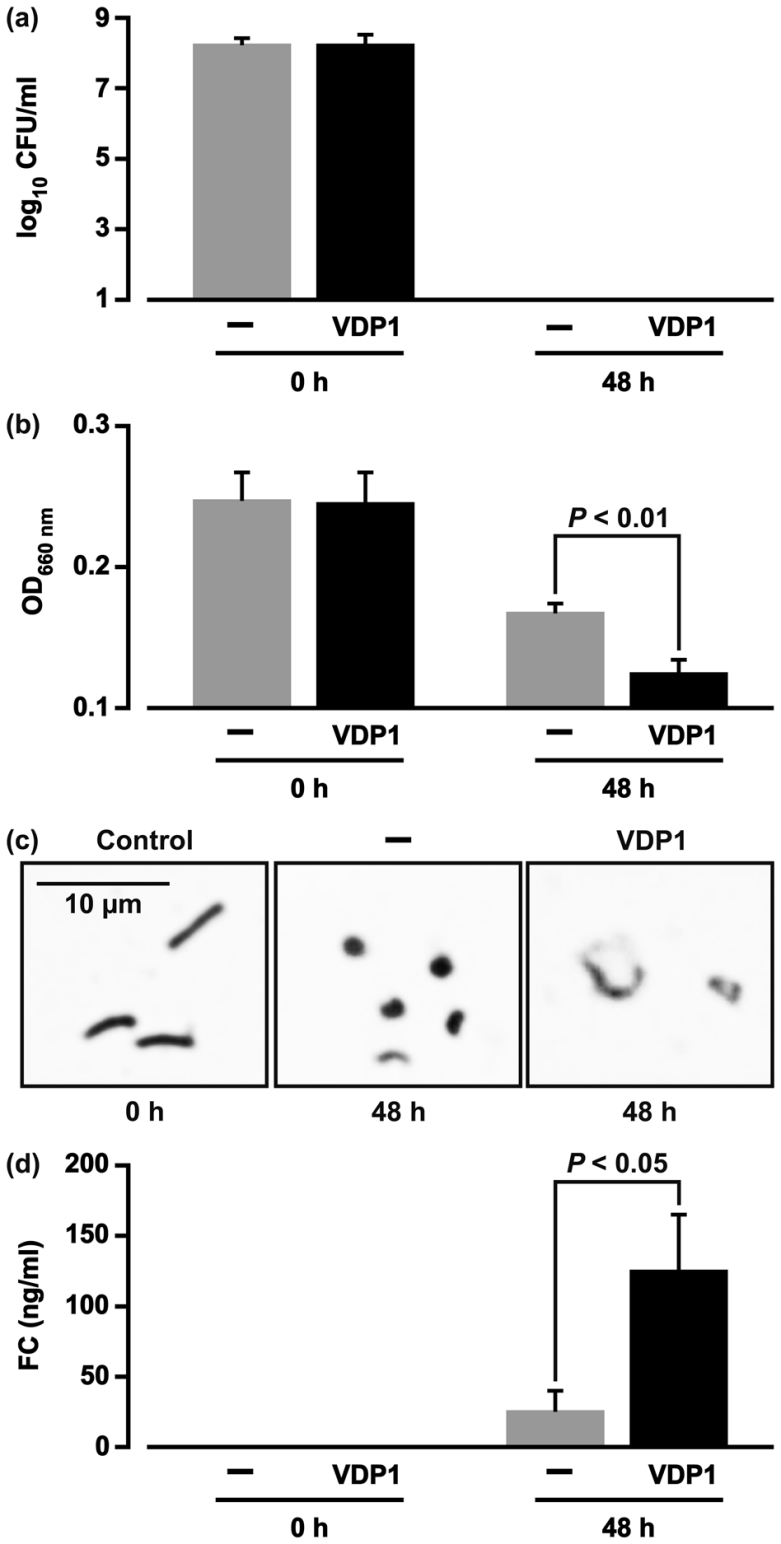
Cell lysis of *H. pylori* induced by the action of VDP1. (a) FC-retaining *H. pylori* was incubated for 48 h in the presence or absence of VDP1 at a 10 μg/ml concentration in PBS containing dMβCD (30 μM) under an anaerobic condition, and the CFUs were counted. Three independent experiments were carried out to calculate the mean CFU ± SD per ml. (b) The OD_660 nm_ of bacterial suspensions was measured after performing the same experiment described in panel (a). The statistical significance of differences in OD_660 nm_ between the bacterial suspensions in the presence and absence of VDP1 was evaluated by the *t*-test based on data obtained from three independent pair-experiments. (c) *H. pylori* cells obtained from the experiment described in panel (a) were microscopically observed. (d) The cell membrane lipids of FC-retaining *H. pylori* were extracted from the bacterial cell supernatant obtained from the same experiment described in panel (a), in order to quantify the FC amounts via the ferrous chloride-sulfuric acid method. The statistical significance of differences in FC amounts detected in the bacterial cell supernatants in the presence and absence of VDP1 was evaluated by the *t*-test based on data obtained from three independent pair-experiments. The “0 h” in panels (a), (b), (c) and (d) indicates the CFU, OD_660 nm_, bacterial shape, and FC amount analyzed immediately before exposure to the anaerobic atmosphere, respectively.

*H. pylori* transforms morphologically from a helical shape to a coccoid shape when exposed to an anaerobic atmosphere^14^. When *H. pylori* altered to the coccoid shape is returned to a microaerophilic condition, the bacterium is incapable of recovering its helical shape or of forming colonies on an agar plate by a conventional culture procedure. To analyze the antibacterial mechanism of VDP1 against *H. pylori*, we performed another set of experiments under anaerobic conditions. When FC-retaining *H. pylori* was incubated for 48 h in the presence or absence of VDP1 (10 μg/ml) in PBS under anaerobic conditions, the CFUs were below the limits of detection regardless of the presence of VDP1 (Fig. 5a). Meanwhile, the OD_660 nm_ of the bacterial suspension was statistically lower in the presence of VDP1 than in its absence (Fig. 5b). To investigate whether the bacterial cells had lysed via the action of VDP1, as this result implied, we observed the cellular shapes in the bacterial suspensions under a microscope. The bacterial cells before the incubation under anaerobic conditions were rod-like in shape (Fig. 5c). Yet in the bacterial suspension incubated without VDP1, the *H. pylori* cells changed to a coccoid shape after 48 h of incubation under anaerobic conditions. Meanwhile, the *H. pylori* cells in the bacterial suspension incubated with VDP1 for 48 h under the same conditions degraded into cellular debris. We next measured the amounts of FC in the cell supernatants of the FC-retaining *H. pylori* incubated for 48 h in the presence or absence of VDP1. The FC in the cell supernatants incubated with VDP1 was statistically more abundant than the FC in the cell supernatants incubated without VDP1 (Fig. 5d). In sum, VDP1 induced a leakage of FC from the cell membranes of *H. pylori*. These results, together with the result of Fig. 4b, indicate that VDP1 binds to the DMPE of *H. pylori* membrane lipid compositions, destabilizes its membrane structures, and ultimately lyses the bacterial cells.

### Chemical structure of VDP1

**Figure 6:**
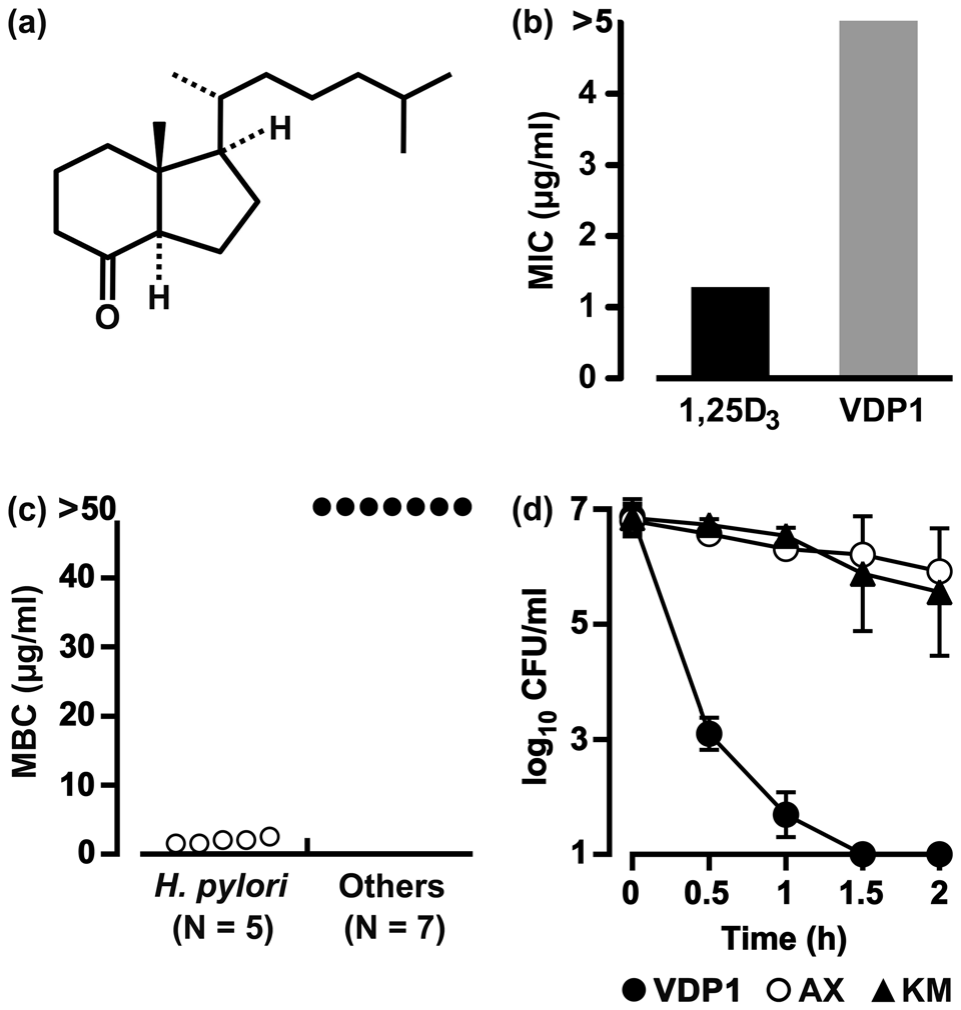
Anti-*H. pylori* activity of synthetic VDP1. (a) Chemical structure of VDP1. (b) The minimum inhibitory concentrations (MICs) of VDP1 and 1α,25-dihydroxyvitamin D_3_ (1,25D_3_) for the FC-retaining *H. pylori* strain NCTC 11638. (c) Eight bacterial species (10^7^ CFU/ml), including five strains of *H. pylori*, were incubated for 24 h in a broth (1.5 ml) containing various concentrations of VDP1 in addition to 30 μM FC. After the incubation, the CFUs were counted to determine the minimum bactericidal concentrations (MBCs) that completely eradicate the 10^6^ CFU of bacteria. Seven bacterial species were used in the experiment: *S. aureus*, *E. coli*, *Salmonella*, *K. pneumoniae*, *P. mirabilis*, *S. marcescens* and *P. aeruginosa*. (d) The FC-retaining *H. pylori* strain NCTC 11638 was incubated for 0.5 to 2 h in the presence of a 2 μg/ml concentration of VDP1, amoxicillin (AX) or kanamycin (KM) in a broth (1.5 ml) containing 30 μM FC, and the CFUs were counted. The mean CFU ± SD per ml was calculated from three independent experiments.

The analysis of both ^1^H-NMR and ^13^C-NMR identified VDP1 with Grundmann's ketone, (1R,3aR,7aR)-1-[(1R)-1,5-dimethylhexyl]octahydro-7a-methyl-4*H*-inden-4-one, which was obtained via the oxidative reaction of vitamin D_3_ catalyzed by ruthenium (Fig. 6a). The NMR spectra of the purified VDP1 were detected as follows: ^1^H NMR (500 MHz, CDCl_3_) δ 2.44 (dd, J = 11.8, 7 Hz, 1H), 2.26 (ddd, J = 14.2, 6.0, 2.0 Hz, 1H), 2.22 (m, 1H), 2.12 (ddd, J = 13.1, 9.6, 2.8 Hz, 1H), 2.00 (m, 1H), 1.90 (m, 2H), 1.72 (m, 1H), 1.52 (m, 3H), 1.42 (m, 2H), 1.34 (m, 2H), 1.30 (m, 1H), 1.14 (m, 3H), 1.04 (m, 1H), 0.95 (d, J = 6.3 Hz, 3H), 0.87 (d, J = 2.3 Hz, 3H), 0.87 (d, J = 2.3 Hz, 3H), 0.64 (s, 3H); ^13^C NMR (125 MHz, CDCl_3_) δ 212.5, 62.2, 56.9, 50.1, 41.2, 39.6, 39.1, 36.2, 35.7, 28.1, 27.7, 24.2, 24.0, 23.0, 22.7, 19.2, 18.9, 12.6. Meanwhile, the NMR spectra of the synthesized VDP1 were detected as follows: ^1^H NMR (500 MHz, CDCl_3_) δ 2.45 (dd, J = 11.8, 7 Hz, 1H), 2.27 (ddd, J = 14.2, 6.0, 2.0 Hz, 1H), 2.22 (m, 1H), 2.12 (ddd, J = 13.1, 9.6, 2.8 Hz, 1H), 2.00 (m, 1H), 1.90 (m, 2H), 1.72 (m, 1H), 1.57 (m, 1H), 1.52 (m, 2H), 1.42 (m, 1H), 1.39 (m, 1H), 1.34 (m, 2H), 1.30 (m, 1H), 1.14 (m, 3H), 1.04 (m, 1H), 0.95 (d, J = 6.3 Hz, 3H), 0.87 (d, J = 2.3 Hz, 3H), 0.87 (d, J = 2.3 Hz, 3H), 0.64 (s, 3H); ^13^C NMR (125 MHz, CDCl_3_) δ 212.4, 62.2, 56.9, 50.1, 41.1, 39.6, 39.1, 36.1, 35.7, 28.1, 27.7, 24.2, 24.0, 23.0, 22.7, 19.2, 18.9, 12.6 (see Supplementary Figs. S1 and S2).

### Antibacterial activity of synthetic VDP1 against *H. pylori* and other bacteria

We next confirmed whether the VDP1 itself was the source of the bactericidal action against *H. pylori*, using a chemical synthetic VDP1 generated from the oxidative reaction of vitamin D_3_. We first examined the minimum inhibitory concentration (MIC) of chemical synthetic VDP1 for *H. pylori* by the agar plate dilution method. The MIC of 1α,25-dihydroxyvitamin D_3_ for the FC-retaining *H. pylori* was 1.25 μg/ml (3 μM). Yet, the MIC of chemical synthetic VDP1 for the same phenotypic *H. pylori* required larger concentration than the 5 μg/ml concentration (>19 μM) (Fig. 6b). In sum, the conventional agar plate dilution method was unsuitable for the determination of MIC of VDP1 for *H. pylori*. Therefore, we adopted the broth dilution method for determination of the minimum bactericidal concentration (MBC) of VDP1 that completely kills the 10^6^ CFU of *H. pylori*. As observed with the purified VDP1, the chemical synthetic VDP1 also exerted antimicrobial action against FC-retaining *H. pylori*. The CFUs of five *H. pylori* strains incubated for 24 h in the presence of synthetic VDP1 at concentrations from 1.5 to 2.5 μg/ml were therefore below the limits of detection (Fig. 6c). Meanwhile, another seven bacteria, namely *E. coli*, *S. aureus*, *P. aeruginosa*, *K. pneumoniae*, *P. mirabilis*, *S. marcescens* and *S. enterica* subsp. *enterica* serovar Typhimurium were entirely unaffected by any antibacterial action of the synthetic VDP1. Even in the presence of VDP1 at a 50 μg/ml concentration, the CFU counts of these bacterial species were comparable to the counts in the absence of VDP1. Thus, *H. pylori* turned out to be tremendously more susceptible to VDP1 than the other bacterial species. This is likely to be explained by the difference in the predominant fatty acid molecules attached to the PE of the respective bacteria: myristic acid is the predominant molecule attached to *H. pylori* PE, while palmitic acid is the predominant molecule attached to the PE of all of the other bacteria except for *S. aureus* that does not possess PE^15^,^16^,^17^,^18^,^19^,^20^,^21^.

### Bactericidal capability of synthetic VDP1 against *H. pylori*

We next assessed the ability of VDP1 to eradicate *H. pylori* compared to amoxicillin and kanamycin, antibiotics that disturb bacterial metabolism. According to measurements of the CFUs of the FC-retaining *H. pylori* along the time axis of incubation in the presence of a 2 μg/ml concentration of VDP1, amoxicillin, or kanamycin, VDP1 conferred a more rapid bactericidal action against *H. pylori* than the other two antibiotics (Fig. 6d). This result demonstrates that VDP1 directly injures the cell membranes of *H. pylori* without necessarily inhibiting the cellular metabolism.

### Influence of synthetic VDP1 on the viability of MKN45 cells and T47D cells

**Figure 7:**
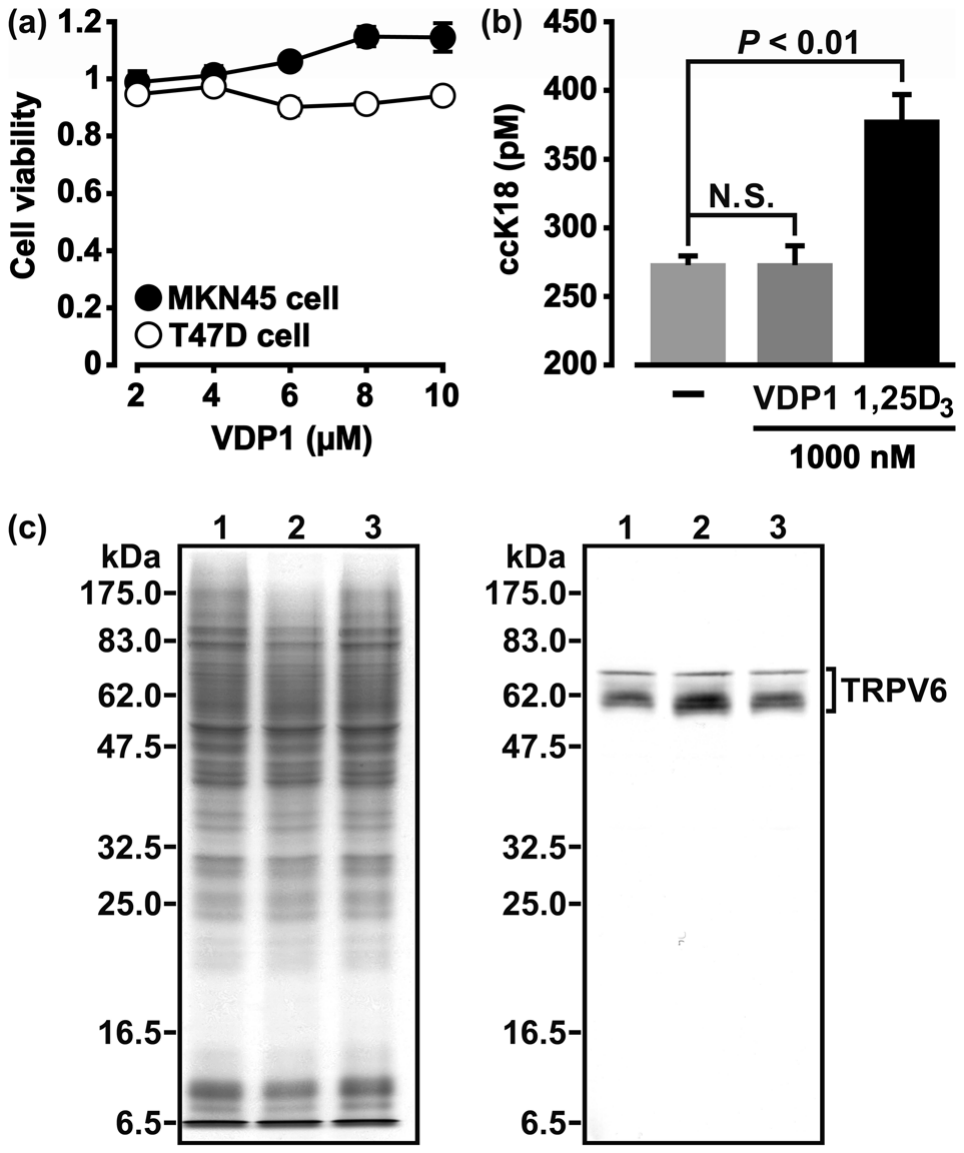
Lack of toxicity and vitamin D_3_-like activity of VDP1 against human cells. (a) MKN45 cells or T47D cells were incubated for 72 h in the presence of VDP1 at the concentration ranges bactericidal to *H. pylori* in order to estimate the cell viability by MTT assay. The cell viabilities obtained from experiments performed in triplicate to calculate the mean value ± SD are denoted by the relative A_540 nm_ in comparison with the value of 1 at the A_540 nm_ of the viability of the cells incubated without VDP1. (b) Caspase-cleaved keratin 18 (ccK18) was detected in the culture supernatant of T47D cells stimulated for 72 h with either VDP1 or 1α,25-dihydroxyvitamin D_3_ (1,25D_3_). The statistical significance of ccK18 amounts between the cells with or without stimulation was evaluated via the *t*-test based on data obtained from three independent pair-experiments. N.S. stands for “no statistical significance”. (c) Transient receptor potential vanilloid type 6 (TRPV6) protein was detected in the lysates of T47D cells stimulated with either VDP1 (1 μM) or 1,25D_3_ (1 μM). The left-hand panel shows the SDS-PAGE profiles of proteins stained by CBB. The right-hand panel shows the TRPV6 protein profiles detected with anti-TRPV6 antibody. The same amounts of cell lysates were applied to SDS-PAGE in the two panels. Lanes 1, 2, and 3 in the two panels are the protein profiles of un-stimulated cells, 1,25D_3_-stimulated cells, and VDP1-stimulated cells, respectively.

We next examined whether the synthetic VDP1 has toxic effects against cell lines of human gastric cancer (MKN45) and breast cancer (T47D). MKN45 cells or T47D cells were incubated for 72 h in the presence of VDP1 at concentrations ranging from 2 to 10 μM, for estimation of the cell viability by MTT assay. VDP1 had no influence on the viability of either MKN45 cells or T47D cells: the cells incubated in the presence of VDP1 at every concentration proliferated to a degree comparable to the same cells incubated without VDP1 (Fig. 7a). This finding confirms that VDP1 is incapable of eliciting toxicity against human cancer cell lines, and it furthermore suggests that any cytotoxic side effects from VDP1 are likely to be extremely weak in human cells.

### Inability of VDP1 to induce a vitamin D_3_-like activation of T47D cells

An 1α,25-dihydroxyvitamin D_3_ induces the formation of a heterodimer consisting of vitamin D receptor (VDR) and retinoid X receptor (RXR) inside the nucleus of the mammalian cell. A recent study has demonstrated that 1α,25-dihydroxyvitamin D_3_ is capable of binding to retinoic acid-related orphan receptors α and γ (RORα and RORγ)^22^. The VDR-RXR coupled with 1α,25-dihydroxyvitamin D_3_ binds to the vitamin D_3_ response elements (VDREs) located 5′-upstream from the target genes, acts as a transcriptional factor, and up-regulates the gene expression of various proteins such as caspases and TRPV6^23^,^24^,^25^,^26^,^27^,^28^,^29^,^30^. Caspases increase the expression levels of caspase-cleaved keratin 18 (ccK18), a cytokeratin associated with cell apoptosis^31^,^32^. In sum, 1α,25-dihydroxyvitamin D_3_ is capable of eliciting increases in the production of ccK18 and TRPV6 proteins in human cells. Incidentally, ROR monomer coupled with 1α,25-dihydroxyvitamin D_3_ interacts with the gene sequences of ROR responsive element (RORE) and inhibits the expression of the target genes^22^. To examine whether synthetic VDP1 exhibits a vitamin D_3_-like action, we stimulated T47D cells for 72 h with either VDP1 or 1α,25-dihydroxyvitamin D_3_ and analyzed the expression levels of ccK18 and TRPV6. The level of ccK18 protein in T47D cells stimulated with 1α,25-dihydroxyvitamin D_3_ was higher than that in cells stimulated with VDP1, while the level of cck18 in T47D cells stimulated with VDP1 was comparable to the level in un-stimulated cells (Fig. 7b). As seen in the experiments with ccK18, the protein band of TRPV6 was denser in T47D cells stimulated with 1α,25-dihydroxyvitamin D_3_ than in cells without stimulation, but the level of TRPV6 protein detected in T47D cells stimulated with VDP1 was comparable to the level of its protein detected in un-stimulated cells (Fig. 7c). These results suggest the possibility that VDP1 is incapable of activating human cells, as was observed with 1α,25-dihydroxyvitamin D_3_.

## Discussion

A recent study by another group has reported that a relatively large amount of the Grundmann's ketone derivative dotted into a paper disk shows the antimicrobial action especially against fungi and Gram-positive bacteria on the agar plate^33^. The above-mentioned study, however, examined neither the antibacterial activity of its Grundmann's ketone derivative against *H. pylori* nor the antimicrobial activity of Grundmann's ketone (VDP1) itself. This study demonstrated the anti-*H. pylori* activity of VDP1 itself, although the MIC of VDP1 for *H. pylori* was incapable of measuring by the conventional agar plate dilution method. The Grundmann's ketone derivative investigated by another group has a positive charge due to the amine in its detergent-like structure and is therefore guessed to diffuse even into the agar plate from the paper disk. Meanwhile, VDP1 is a high non-polar ketone compound. On this basis, VDP1 may be difficult to extensively diffuse into the agar plate. Therefore, the agar plate dilution method seems to be unsuitable for the decision of MICs of VDP1 for the bacteria. Incidentally, given that the polarity of 1α,25-dihydroxyvitamin D_3_ is higher than that of VDP1 due to the three hydroxyl groups in its seco-steroid frame, we can assume that 1α,25-dihydroxyvitamin D_3_ diffuses more extensively than VDP1 into the agar plate. We will need to synthesize such new VDP1 derivatives as reported by another group^33^ to compare the agar diffusion of the VDP1 derivatives with that of VDP1 itself.

An earlier study by another group has shown that elderly women who had been supplied a vitamin D_3_ analogue had a lower rate of *H. pylori* infection than elderly women without the analogue, though it was unclear whether the analogue had exerted a direct antibacterial action against the *H. pylori*^34^. The present study demonstrated that intact compounds of both 25-hydroxyvitamin D_3_ and 1α,25-dihydroxyvitamin D_3_ exerted antibacterial actions against *H. pylori* without FC. Our findings also confirmed that the two vitamin D_3_ metabolites are capable of eradicating the FC-retaining *H. pylori* (data not shown). Hence, we have discovered that 25-hydroxyvitamin D_3_ and 1α,25-dihydroxyvitamin D_3_ confer a novel biological action that is clearly distinct from the hormonal action on mammals^35^,^36^,^37^,^38^,^39^,^40^,^41^,^42^,^43^. The colonization of the human stomach by *H. pylori* is accomplished in childhood. We know this because the gut immunities of children are still incomplete^44^,^45^. Serum 25-hydroxyvitamin D_3_ levels in childhood vary between the pediatric populations investigated, and children with extremely low 25-hydroxyvitamin D_3_ levels in serum are commonly observed^46^,^47^. This may suggest that low 25-hydroxyvitamin D_3_ levels in serum are correlated with the incidence of *H. pylori*-infection in childhood. The goal for future studies will be to elucidate the detailed antibacterial mechanism of 25-hydroxyvitamin D_3_ and 1α,25-dihydroxyvitamin D_3_ against *H. pylori* and to analyze the epidemiological relationship between serum 25-hydroxyvitamin D_3_ levels and *H. pylori*-infection in children.

This study elucidated the bactericidal effect of VDP1 (Grundmann's ketone) against *H. pylori* *in vitro*. In the next stage, experiments using animal models will be essential to estimate the treatment effect of VDP1 on *H. pylori*-infected hosts and to investigate the side effects of the vitamin D_3_ decomposition product on mammals. We will also need to examine whether other bacterial species from the genus *Helicobacter* are susceptible to the bactericidal action of VDP1.

## Methods

### Bacterial culture

Bacteria were cultured in a pleuropneumonia-like organisms (PPLO) broth (Difco Laboratories, Detroit MI) at 37°C in a Concept 400 microaerophilic chamber (10% CO_2_, 5% O_2_ and 85% N_2_) (Ruskinm Technology, Leeds, UK).

### Colony-forming units (CFUs)

Bacterial suspensions (100 μl) prepared in 10-fold serial dilutions using a PPLO broth were spread on brain-heart infusion agar (Difco Laboratories) plates containing 5% horse serum (Gibco, Auckland, NZ), and cultured for 1 week in the microaerophilic chamber. The bacterial colonies grown were counted on the agar plates spread with appropriate bacterial cell diluents to calculate the log_10_ CFU/ml.

### Bacterial cell density (OD_660 nm_)

Bacterial suspensions (200 μl) were measured at a wavelength of 660 nm. PBS was used as the medium of bacterial suspension.

### Microscopic observation

Bacterial cells suspended in PBS (10 μl) were applied on a glass slide, dried at room temperature, and stained for 10 min with Coomassie brilliant blue (CBB) solution (0.05% CBB, 9% acetate, 45.5% methanol).

### Analysis of the hydrophobic compounds

Solvents of chloroform-methanol (2:1) were added at 5-fold volume to an aqueous solution in a glass bottle and intensely shaken. The mixed medium of water and organic solvents was re-separated into two liquid phases via incubation for 24 h at 4°C. After the upper liquid phase was removed, the solvents of the lower liquid phase were vaporized under nitrogen airflow or via use of a rotary evaporator to obtain the hydrophobic compounds. To analyze the hydrophobic compounds via thin-layer chromatography (TLC), the specimens dissolved in a chloroform-methanol (2:1) solution (40 μl) were dotted onto a Silica Gel 60 plate (Merck, Darmstadt, Germany) and fractionated on the plate surface with the developing solvents. After the TLC, the plate was sprayed with a 60% sulfuric acid solution and heated at 120 to 180°C to visualize the spots of the components on the TLC plate surface.

### Quantification of free cholesterol (FC)

Lipid specimens dissolved in acetic acid solution (600 μl) were added to a ferrous chloride-sulfuric acid reagent [phosphoric acid-sulfuric acid (2:25) solution containing 0.2% FeCl_2_-6H_2_O] solution (400 μl), thoroughly stirred, and incubated for 15 min at room temperature. After color reaction and cooling, the absorbance (A_550 nm_) of the mixed solution (200 μl) was measured at a wavelength of 550 nm. The amounts of FC in the specimens were quantified based on an FC standard curve calculated from the A_550 nm_ (y axis) and known FC amounts (x axis).

### Preparation of a phosphatidylethanolamine (PE) vesicle suspension containing CBB

A powder (15 mg) of dimyristoyl PE (DMPE: Sigma-Aldrich Inc., MO) or dipalmitoyl PE (DPPE: Sigma-Aldrich Inc.) dispersed into a 50 mM Tris (pH 7.5) buffer (4 ml) containing 150 mM sucrose was sonicated for 6 to 8 h in a cold sonicator of the bucket type. After confirmation of the PE vesicle formation via microscopic observation, the PE vesicles were washed three times with a 50 mM Tris (pH 7.5) buffer (1 ml) via centrifugation (10000 × g, 5 min), suspended in the same buffer (2 ml) containing 0.1% CBB, sonicated for 1 h, and shaken overnight at 4°C. After the PE vesicle suspension containing CBB was washed three times with a 50 mM Tris (pH 7.5) buffer via centrifugation (10000 × g, 5 min), a PE vesicle suspension of the same buffer (100 μl) was adjusted to a value of 2 in the OD_660 nm_ and stored at −20°C until use in experiments.

### Vitamin D_3_ species and others

Vitamin D_3_ from Wako Pure Chemical Industries Ltd. (Tokyo, Japan), circulating-form of vitamin D_3_ (25-hydroxyvitamin D_3_) from Enzo Life Sciences Inc. (NY), or activated form of vitamin D_3_ (1α,25-dihydroxyvitamin D_3_) from Cayman Chemical Co. (MI) was dissolved in ethanol at a 5 mM concentration and stored as stock solution at −80°C. The 0.2% concentration of ethanol had no influence on the survival of either bacteria or human cells. Two antibiotics, namely, kanamycin sulfate from EMD Biosciences Inc. (CA), and amoxicillin from Sigma-Aldrich Inc., were respectively dissolved in distilled water at 1 mg/ml concentrations and stored at 4 or −30°C as stock solutions.

### Assay of bacterial growth

Bacteria were shaken in the presence of various concentrations of antibacterial substances in either PPLO broth alone (1.5 ml) or PPLO broth (1.5 ml) containing a 30 μM concentration of FC in the microaerophilic chamber, and the CFUs were measured. Five *H. pylori* strains were investigated: NCTC 11638, ATCC 43504, 26695, and clinical isolates A-13 and A-19. Two phenotypes of *H. pylori* were used: FC-free cells and FC-retaining cells. The FC-free *H. pylori* were prepared by the culture procedure without FC. The FC-retaining *H. pylori* were prepared by culturing for three generations in the presence of FC (Wako Pure Chemical Industries Ltd.) at a 30 μM concentration. Seven typical bacterial strains maintained for many years in our laboratory were also used in this assay: *Escherichia coli*, *Staphylococcus aureus*, *Pseudomonas aeruginosa*, *Klebsiella pneumoniae*, *Proteus mirabilis*, *Serratia marcescens*, and *Salmonella enterica* subsp. *enterica* serovar Typhimurium.

### Non-biological degradation of vitamin D_3_ species

Each of the vitamin D_3_ species (150 nmol) was stirred for 24 to 48 h in distilled water (1.5 ml) at 70°C, recovered by the organic solvent distribution method described in the section on the analysis of hydrophobic compounds, subjected to TLC with a chloroform-acetone-methanol (9:1:1) solvent system to analyze its degradation. Next, the decomposition products obtained from each vitamin D_3_ species were dispersed into the bacterial suspensions (1.5 ml) of the *H. pylori* strain NCTC 11638 and were shaken for 2 h in the microaerophilic chamber to count the CFUs.

### Purification of vitamin D_3_ decomposition products 1 and 2 (VDP1 and VDP2)

Vitamin D_3_ (100 mg) was dispersed into distilled water (100 ml) and incubated for 1 week at 70°C to obtain its decomposition products (VDPs), and the VDPs were then recovered via the organic solvent distribution method and dissolved in a chloroform solvent (1 ml). After the VDP solution was applied onto an Iatrobead 6RS-8060 (Mitsubishi Kagaku Iatron Inc., Tokyo, Japan) column (1-cm diameter, 5-cm height) activated with a chloroform solvent, the VDPs were divided into four fractions respectively eluted with a chloroform solvent (10 ml), chloroform-acetone (9:1) solvents (10 ml), chloroform-acetone (7:3) solvents (10 ml), and chloroform-acetone (4:6) solvents (10 ml), and dried using a rotary evaporator.

Next, the VDP fraction eluted with the chloroform solvent was further divided into nine aliquots (500 μl/tube) eluted with diethylether-chloroform (7:3) solvents using the same Iatrobead 6RS-8060-column, and dried using a rotary evaporator. Two-dimensional TLC was carried out to confirm VDP1 and VDP2 using hexane-diethylether (6:4) solvents and then hexane-ethylacetate (10:1) solvents.

Next, the aliquots containing VDP1 and VDP2 were collected and applied onto a Purif Pack SI30 Flash column (size 20, 2-cm diameter, 6-cm height) (Shoko Scientific, Kanagawa, Japan). The VDP1 and VDP2 were eluted from the column at a flow velocity of 15 ml/min using a hexane solvent containing ethylacetate with concentration gradient, and the chromatograms of eluates were monitored by measuring the absorbance (A_254 nm_) at a wavelength of 254 nm (ChemGenesis Inc., Tokyo, Japan).

Finally, VDP1 and VDP2 were purified by high-performance liquid chromatography (HPLC) using a GL Science Inertsil SIL 100A column (2-cm diameter, 25-cm height; GL Sciences Inc., Tokyo, Japan). The HPLC was carried out at a flow velocity of 20 ml/min using a hexane solvent containing 3% ethylacetate by monitoring the chromatograms of eluates measured with A_254 nm_ (ChemGenesis Inc.). The purity of VDP1 and VDP2 after the HPLC application was confirmed by TLC analysis with a hexane-ethylacetate (10:1) solvent system. The purified VDP1 and VDP2 were dissolved in ethanol solution and stored at −80°C until use in experiments.

### Analysis of the interaction of VDP1 and VDP2 with the PE vesicles

A PE vesicle suspension (50 μl) prepared by the method described in the section on preparing the phosphatidylethanolamine (PE) vesicle suspension containing CBB was added into a 50 mM Tris (pH 7.5) buffer (1.45 ml) containing 15 μg of either VDP1 or VDP2, and shaken for 2 h at 37°C. After the PE vesicles were removed via centrifugation (10000 × g, 5 min), the absorbance of the supernatant (200 μl) was measured at a wavelength of 590 nm to detect the elution of CBB from the PE vesicles.

### Analysis of the anti-*H. pylori* mechanism of VDP1

A bacterial suspension (200 μl) of *H. pylori* strain NCTC 11638 was cultured for 24 h with shaking in a PPLO broth (10 ml) containing a 30 μM concentration of FC and 10 μM concentration of 2,6-di-*O*-methyl-β-cyclodextrin (dMβCD: Sigma-Aldrich Inc.) in the microaerophilic chamber. This culture procedure was repeated three times to prepare the FC-retaining *H. pylori* cells. After three washes with PBS by centrifugation (10000 × g, 5 min), the bacterial cells (10^9^ CFU) were suspended in PBS (5 ml) containing dMβCD (30 μM) and shaken for 48 h in the presence or absence of VDP1 (10 μg/ml) at 37°C in a box filled with anaerobic gas using an AnaeroPack (Mitsubishi Gas Chemical Co., Inc., Tokyo, Japan). The CFUs were counted, the OD_660 nm_ of the bacterial suspension (200 μl) was measured and the bacterial cells stained with CBB were observed. After filtrating the bacterial suspension (4 ml) through a GDXS 25 syringe filter (Whatman, Buckinghamshire, UK), the lipids from the *H. pylori* cells were extracted from the filtrates by the organic solvent distribution method to measure the FC amounts released from the cell membranes.

### Analysis of the chemical structure of VDP1

Nuclear magnetic resonance (NMR) spectra were recorded on 500 MHz for ^1^H and 125 MHz for ^13^C in the indicated solvent using a Model ECA-500 (JEOL Ltd., Tokyo, Japan). Chemical shifts were reported in parts per million (ppm) relative to the signal (0 ppm) for internal tetramethylsilane in CDCl_3_. ^1^H-NMR spectral data were reported as follows: CDCl_3_ (7.26 ppm). ^13^C-NMR spectral data were reported as follows: CDCl_3_ (77.0 ppm).

### Chemical synthesis of VDP1

To the stir solution of a catalytic amount of RuCl_3_ and NaIO_4_ (1.22 mmol) in ethylacetate-acetonitrile-water (3:3:1) at 0°C, vitamin D_3_ (0.78 mmol) from Wako Pure Chemical Industries Ltd. was added. After stirred for 5 h at room temperature, the reaction mixture was diluted with hexane, quenched with 10% aq. Na_2_S_2_O_3_, and the aqueous layer was extracted with two portions of hexane. The combined extract was washed with brine and evaporated *in vacuo*. The residue was chromatographed on a silica gel (Merck) with hexane-ethylacetate (4:1) to give VDP1 (Grundmann's ketone) from the aqueous layer (see Supplementary Fig. S3)^48^,^49^,^50^. The mass spectrum of the synthetic VDP1 was detected via the gas chromatography-mass spectrometry (GC-MS) using a JMS-Q1000GCMkII system (JEOL Ltd.) in order to be compared to the mass spectrum of the VDP1 described in the section on purifying the vitamin D_3_ decomposition products 1 and 2 (VDP1 and VDP2) (see Supplementary Fig. S4).

### Measurement of minimum inhibitory concentrations (MICs)

Various concentrations of specimens were added to the 30 μM FC-dispersed PPLO agar plates. The bacterial suspension (10 μl) of FC-retaining *H. pylori* (10^8^ CFU/ml) was dropped onto the agar plates and incubated for 1 week under microaerobic conditions at 37°C. After incubation, the MICs of the specimens for *H. pylori* were measured by confirming the growth of colonies on the agar plates. Two independent experiments were performed to determine the MICs.

### Analysis of cytotoxicity of VDP1

Human breast cancer T47D cells from DS Pharma Biomedical Co., Ltd. (Osaka, Japan) and human gastric cancer MKN45 cells from GeneticLab Co., Ltd. (Sapporo, Japan) were maintained in an RPMI 1640 (10% FCS-RPMI) medium (Sigma-Aldrich Inc.) supplemented with 10% heat-inactivated fetal calf serum (FCS: Gibco), 10 mM HEPES, 2 mM L-glutamine, 100 U/ml penicillin, 100 μg/ml streptomycin and 0.2% NaHCO_3_ in a humidified chamber at 37°C filled with a 5% CO_2_ gas. After the cells were washed once with PBS and adjusted to 10^5.3^ cell/ml using an RPMI 1640 medium supplemented with 2.5% FCS in place of 10% FCS, the cell suspension (100 μl) was inoculated into the same medium (100 μl) containing VDP1 at various concentrations and incubated for 72 h at 37°C in the 5% CO_2_ incubator. A 20 μl volume of MTT (Sigma-Aldrich Inc.) reagent (5 mg/ml in PBS) was added into the cell cultures in the final 4 h of the 72-h incubation. After the cell supernatant was removed, the cells were lysed in an isopropanol solution (200 μl) containing 5% formic acid in order to solubilize the formazan blue crystals produced from the viable cells, and the absorbance of formazan blue solution (150 μl) was measured at a wavelength of 540 nm.

### Detection of caspase-cleaved keratin 18 (ccK18)

T47D cells adjusted to 10^5.5^ cell/ml using 10% FCS-RPMI medium (1 ml) were incubated for 72 h in the presence of various concentrations of either VDP1 or 1α,25-dihydroxyvitamin D_3_ at 37°C in the 5% CO_2_ incubator, and the culture supernatants were then recovered. The ccK18 in the culture supernatants was detected using a M30 CytoDeath ELISA kit (Peviva AB, Stockholm, Sweden) according to the manufacturer's instructions.

### Detection of transient receptor potential vanilloid type 6 (TRPV6) protein

After T47D cells were cultured by the same method described in the section on detecting caspase-cleaved keratin 18 (ccK18), the culture supernatant was removed and the cells were lysed in a 50 mM Tris (pH 6.8) buffer (100 μl) containing 5% glycerol, 0.002% bromophenol blue (BPB), 2% sodium dodecyl sulfate (SDS), and 40 mM dithiothreitol (DTT). Cell lysates (5 μl) were electrophoresed in a 0.1% SDS-12.5% polyacrylamide gel to separate the proteins (SDS-PAGE). The proteins were then stained with CBB solution or transferred onto a Clear Blot P-membrane (p-membrane: ATTO Co., Tokyo, Japan) activated with a Tris-glycine buffer (100 mM Tris, 192 mM glycine, 5% methanol) using a blotting device (ATTO Co.). The p-membrane was shaken overnight in 20 mM Tris (pH 7.6) buffered saline (TBS) containing 1% skim milk at 25°C, and shaken for 2 h in the presence of anti-TRPV6 rabbit IgG polyclonal antibody (Santa Cruz Biotechnology Inc., CA) in a reaction buffer (0.1% skim milk, 0.1% Tween 20 in TBS) at 25°C. The p-membrane was then washed three times with Tween 20-TBS (0.1% Tween 20 in TBS) and shaken for 2 h at 25°C in the presence of horseradish peroxidase (HRP) conjugate anti-rabbit IgG polyclonal antibody (Cell Signaling Technology Inc., MA) in the reaction buffer. After further washes of the p-membrane in Tween 20-TBS (three tines), TBS (twice), and distilled water (once), the enzymatic substrate reaction of HRP was developed on the p-membrane in a 100 mM Tris (pH 7.5) buffer containing 0.04% H_2_O_2_ and 0.027% DAB (3,3′-diaminobenzidine-tetra-hydrochloride-dihydrate), and the reaction was stopped by a wash in water.

## Author Contributions

H.S., S.H. and Y.H. conceived the study, and H.S. constructed the experiment methods and wrote a large part of the manuscript. K.H. and A.A. carried out the bioassay of chemical compounds together with H.S., and K.W. and H.M. synthesized and purified the chemical compound and analyzed it together with T.T. All authors reviewed the manuscript.

## Supplementary Material

Supplementary Information Supplementary Information

## References

[b1] Marshall B. & Warren J. R. Unidentified carved bacilli on gastric epithelium in active chronic gastritis. Lancet 1, 1273–1274 (1983). 6134060

[b2] Fukase K. *et al.* Effect of eradication of *Helicobacter pylori* on incidence of metachronous gastric carcinoma after endoscopic resection of early gastric cancer: an open-label randomized controlled trial. Lancet 372, 392–397 (2008). 18675689 10.1016/S0140-6736(08)61159-9

[b3] Stolte M. *et al.* Helicobacter and gastric MALT lymphoma. Gut 50, III19-III24 (2002). 10.1136/gut.50.suppl_3.iii19 PMC1867678 11953328

[b4] Kobayashi I. *et al.* Changing antimicrobial susceptibility epidemiology of *Helicobacter pylori* strains in Japan between 2002 and 2005. J. Clin. Microbiol. 45, 4006–4010 (2007). 17942652 10.1128/JCM.00740-07 PMC2168569

[b5] Francesco V. De. *et al.* Worldwide *H. pylori* antibiotic resistance: a systematic review. J. Gastrointestin. Liv. Dis. 19, 409–414 (2010). 21188333

[b6] Shimomura H., Hosoda K., Hayashi S., Yokota K. & Hirai Y. Phosphatidylethanolamine of *Helicobacter pylori* functions as a steroid-binding lipid in the assimilation of free cholesterol and 3β-hydroxyl steroids into the bacterial cell membrane. J. Bacteriol. 194, 2658–2667 (2012). 22408164 10.1128/JB.00105-12 PMC3347179

[b7] Amgalanbaatar A. *et al.* Antibacterial activity of a novel synthetic progesterone species carrying a linoleic acid molecule against *Helicobacter pylori* and the hormonal effect of its steroid on a murine macrophage-like cell line. J. Steroid Biochem. Mol. Biol. 140, 17–25 (2014). 24189541 10.1016/j.jsbmb.2013.10.023

[b8] Hosoda K., Shimomura H., Hayashi S., Yokota K. & Hirai Y. Steroid hormones as bactericidal agents to *Helicobacter pylori*. FEMS Microbiol. Lett. 318, 68–75 (2011). 21306429 10.1111/j.1574-6968.2011.02239.x

[b9] Slominski A. T. *et al.* The role of CYP11A1 in the production of vitamin D metabolites and their role in the regulation of epidermal functions. J. Steroid Biochem. Mol. Biol. 144, 28–39 (2014). 24176765 10.1016/j.jsbmb.2013.10.012 PMC4002668

[b10] Grady L. T. & Thakker K. D. Stability of solid drugs: degradation of ergocalciferol (vitamin D_2_) and cholecalciferol (vitamin D_3_) at high humidities and elevated temperatures. J. Pharm. Sci. 69, 1099–1102 (1980). 6251199 10.1002/jps.2600690932

[b11] Shimomura H. *et al.* Steroids mediate resistance to the bactericidal effect of phosphatidylcholines against *Helicobacter pylori*. FEMS Microbiol. Lett. 301, 84–94 (2009). 19843309 10.1111/j.1574-6968.2009.01807.x

[b12] McGee D. J. *et al.* Cholesterol enhances *Helicobacter pylori* resistance to antibiotics and LL37. Antimicrob. Agents Chemother. 55, 2897–2904 (2011). 21464244 10.1128/AAC.00016-11 PMC3101455

[b13] Trainor E. A., Horton K. E., Savage P. E., Testerman T. L. & McGee D. J. Role of the HefC efflux pump in *Helicobacter pylori* cholesterol-dependent resistance to ceragenins and bile salts. Infect. Immun. 79, 88–97 (2011). 20974830 10.1128/IAI.00974-09 PMC3019907

[b14] Shimomura H., Hayashi S., Yokota K., Oguma K. & Hirai Y. Alteration in the composition of cholesteryl glucosides and other lipids in *Helicobacter pylori* undergoing morphological change from spiral to coccoid form. FEMS Microbiol. Lett. 237, 407–413 (2004). 15321690 10.1016/j.femsle.2004.07.004

[b15] Shokri A. & Larsson G. Characterisation of the *Escherichia coli* membrane structure and function during fed batch cultivation. Microb. Cell Fact. 3, 9 (2004). 15282031 10.1186/1475-2859-3-9 PMC514524

[b16] Dunnick J. K. & O'Leary W. M. Correlation of bacterial lipid composition with antibiotic resistance. J. Bacteriol. 101, 892–900 (1970). 4314544 10.1128/jb.101.3.892-900.1970 PMC250407

[b17] Amine A., Saloua K. S., Mouadh M., Alya E. I. M. & Ahmed M. [The absence of the “GATC-binding protein SeqA” affects DNA replication in *Salmonella enterica* serovar Typhimurium]. DNA replication and related cellular processes [Kusic-Tisma J. (ed.)] [283–300] (Rijeka, Croatia, 2011).

[b18] Gmeiner J. & Martin H. H. Phospholipid and lipopolysaccharide in *Proteus mirabilis* and its stable protoplast L-form. Eur. J. Biochem. 67, 487–494 (1976). 786631 10.1111/j.1432-1033.1976.tb10714.x

[b19] Zalkin H., Law J. H. & Goldfine H. Enzymatic synthesis of cyclopropane fatty acids catalyzed by bacterial extracts. J. Biol. Chem. 238, 1242–1248 (1963). 14003136

[b20] Kearns D. B., Robinson J. & Shimkets L. J. *Pseudomonas aeruginosa* exhibits directed twitching motility up phosphatidylethanolamine gradients. J. Bacteriol. 183, 763–767 (2001). 11133973 10.1128/JB.183.2.763-767.2001 PMC94935

[b21] Beining P. R., Huff E., Prescott B. & Theodore T. S. Characterization of the lipids of mesosomal vesicles and plasma membranes from *Staphylococcus aureus*. J. Bacteriol. 121, 137–143 (1975). 1116984 10.1128/jb.121.1.137-143.1975 PMC285623

[b22] Slominski A. T. *et al.* RORα and RORγ are expressed in human skin and serve as receptors for endogenously produced noncalcemic 20-hydroxy- and 20,23-dihydroxyvitamin D. FASEB J. Res. Commun. 28, 2775–2789 (2014). 10.1096/fj.13-242040 PMC4062828 24668754

[b23] Mathiasen I. S., Lademann U. & Jäättelä M. Apoptosis induced by vitamin D compounds in breast cancer cells is inhibited by Bcl-2 but does not involve known caspases or p53. Cancer Res. 59, 4848–4856 (1999). 10519395

[b24] Bolanz K. A., Hediger M. A. & Landowski C. P. The role of TRPV6 in breast carcinogenesis. Mol. Cancer Ther. 7, 271–279 (2008). 18245667 10.1158/1535-7163.MCT-07-0478

[b25] Bolanz K. A., Kovacs G. G., Landowski C. P. & Hediger M. A. Tamoxifen inhibits TRPV6 activity via estrogen receptor-independent pathways in TRPV6-expressing MCF-7 breast cancer cells. Mol. Cancer Res. 7, 2000–2010 (2009). 19996302 10.1158/1541-7786.MCR-09-0188

[b26] Baker A. R. *et al.* Cloning and expression of full-length cDNA encoding human vitamin D receptor. Proc. Natl. Acad. Sci. USA 85, 3294–3298 (1988). 2835767 10.1073/pnas.85.10.3294 PMC280195

[b27] Haussler M. R. *et al.* The vitamin D hormone and its nuclear receptor: molecular actions and disease states. J. Endocrinol. 154, 557–573 (1997). 9379138

[b28] Thompson P. D., Jurutka P. W., Haussler C. A., Whitfield G. K. & Haussler M. R. Heterodimeric DNA binding by the vitamin D receptor and retinoid X receptors is enhanced by 1,25-dihydroxyvitamin D_3_ and inhibited by 9-*cis*-retinoic acid. J. Biol. Chem. 273, 8483–8491 (1998). 9525962 10.1074/jbc.273.14.8483

[b29] Carlberg C. Mechanisms of nuclear signaling by vitamin D_3_ interplay with retinoid and thyroid hormone signaling. Eur. J. Biochem. 231, 517–527 (1995). 7649150

[b30] Schräder M., Nayeri S., Kahlen J. P., Müller K. M. & Carlberg C. Natural vitamin D_3_ response elements formed by inverted palindromes: polarity-directed ligand sensitivity of vitamin D_3_ receptor-retinoid X receptor heterodimer-mediated transactivation. Mol. Cell Biol. 15, 1154–1161 (1995). 7862109 10.1128/mcb.15.3.1154 PMC230337

[b31] Leers M. P. *et al.* Immunocytochemical detection and mapping of a cytokeratin 18 neo-epitope expressed during early apoptosis. J. Pathol. 187, 567–572 (1999). 10398123 10.1002/(SICI)1096-9896(199904)187:5<567::AID-PATH288>3.0.CO;2-J

[b32] Schutte B. *et al.* Keratin 8/18 breakdown and reorganization during apoptosis. Exp. Cell Res. 297, 11–26 (2004). 15194421 10.1016/j.yexcr.2004.02.019

[b33] Krojer M., Keller M. & Bracher F. 7-Aza-des-A-steroids with antimicrobial and cytotoxic activity. Sci. Pharm. 81, 329–338 (2013). 23833707 10.3797/scipharm.1303-03 PMC3700069

[b34] Kawaura A. *et al.* Inhibitory effect of long term 1α-hydroxyvitamin D_3_ administration on *Helicobacter pylori* infection. J. Clin. Biochem. Nutr. 38, 103–106 (2006).

[b35] Gombart A. F., Borregaard N. & Koeffler H. P. Human cathelicidin antimicrobial peptide (CAMP) gene is a direct target of the vitamin D receptor and is strongly up-regulated in myeloid cells by 1,25-dihydroxyvitamin D_3_. FASEB J. Res. Commun. 19, 1067–1077 (2005). 10.1096/fj.04-3284com 15985530

[b36] Meyer M. B., Watanuki M., Kim S., Shevde N. K. & Pike J. W. The human transient receptor potential vanilloid type 6 distal promoter contains multiple vitamin D receptor binding sites that mediate activation by 1,25-dihydroxyvitamin D_3_ in intestinal cells. Mol. Endocrinol. 20, 1447–1461 (2006). 16574738 10.1210/me.2006-0031

[b37] Song Y. *et al.* Calcium transpoter 1 and epithelial calcium channel messenger ribonucleic acid are differentially regulated by 1,25-dihydroxyvitamin D_3_ in the intestine and kidney of mice. Endocrinol. 144, 3885–3894 (2003). 10.1210/en.2003-0314 12933662

[b38] Christakos S., Gabrielides C. & Rhoten W. B. Vitamin D-dependent calcium binding proteins: chemistry, distribution, functional consideration, and molecular biology. Endocrine Rev. 10, 3–26 (1989). 2666110 10.1210/edrv-10-1-3

[b39] Demay M. B., Kierman M. S., DeLuca H. F. & Kronenberg H. M. Sequences in the human parathyroid hormone gene that bind the 1,25-dihydroxyvitamin D_3_ receptor and mediate transcriptional repression in response to 1,25-dihydroxyvitamin D_3_. Proc. Natl. Acad. Sci. USA 89, 8097–8101 (1992). 1325645 10.1073/pnas.89.17.8097 PMC49863

[b40] Shimada T. *et al.* FGF-23 is a potent regulator of vitamin D metabolism and phosphate homeostasis. J. Bone Mineral Res. 19, 429–435 (2004). 10.1359/JBMR.0301264 15040831

[b41] Kolek O. I. *et al.* 1α,25-dihydroxyvitamin D_3_ upregulates FGF23 gene expression in bone: the final link in a renal-gastrointestinal-skeletal axis that controls phosphate transport. Am. J. Physiol. Gastrointest. Liv. Physiol. 289, G1036–G1042 (2005). 10.1152/ajpgi.00243.2005 16020653

[b42] Yamamoto R. *et al.* 1α,25-dihydroxyvitamin D_3_ acts predominantly in mature osteoblasts under conditions of high extracellular phosphate to increase fibroblast growth factor 23 production in vitro. J. Endocrinol. 206, 279–286 (2010). 20530653 10.1677/JOE-10-0058 PMC2917591

[b43] Noda M., Vogel R. L., Craig A. M., Prahl J. & DeLuca H. Identification of a DNA sequence responsible for binding of 1,25-hydroxyvitamin D_3_ receptor and 1,25-hydroxyvitamin D_3_ enhancement of mouse secreted phosphoprotein 1 (*Spp-1* or osteopontin) gene expression. Proc. Natl. Acad. Sci. USA 87, 9995–9999 (1990). 2175918 10.1073/pnas.87.24.9995 PMC55301

[b44] Asaka M. *et al.* Atrophic gastritis and intestinal metaplasia in Japan: results of a large multicenter study. Helicobacter 6, 294–299 (2001). 11843961 10.1046/j.1523-5378.2001.00042.x

[b45] Kobayashi T. *et al.* Trends in the incidence of gastric cancer in Japan and their associations with *Helicobacter pylori* infection and gastric mucosal atrophy. Gas. Cancer 7, 233–239 (2004). 10.1007/s10120-004-0297-0 15616771

[b46] Brehm J. M. *et al.* Serum vitamin D levels and markers of severity of childhood asthma in Costa Rica. Am. J. Respir. Crit. Care Med. 179, 765–771 (2009). 19179486 10.1164/rccm.200808-1361OC PMC2675563

[b47] Mansbach J. M., Ginde A. A. & Camargo, Jr C. A. Serum 25-hydroxyvitamin D levels among US children aged 1 to 11 years: do children need more vitamin D? Pediatrics 124, 1404–1410 (2009). 19951983 10.1542/peds.2008-2041 PMC3765249

[b48] Plietker B. & Niggemann M. RuCl_3_/CeCl_3_/NaIO_4_: a new bimetallic oxidation system for the mild and efficient dihydroxylation of unreactive olefins. J. Org. Chem. 70, 2402–2405 (2005). 15760243 10.1021/jo048020x

[b49] Windaus A. & Grundmann W. Über die konstitution des vitamins D_2_. II. Justus Liebigs Annalen der Chemie. 524, 295–299 (1936).

[b50] Sicinski R. R. & DeLuca H. F. Ruthenium tetroxide oxidation of Grundmann's ketone derivative from vitamin D_3_. Bioor. Med. Chem. Lett. 5, 159–162 (1995).

