# Supplementary material for: Identification and characterization of a vitamin D3 decomposition product bactericidal against Helicobacter pylori
Source: Sci Rep. 2015 Mar 9;5:8860. doi: 10.1038/srep08860 (PMC4352922; doi:10.1038/srep08860)
Supplement: Supplementary Information [file srep08860-s1.pdf]

Identification and characterization of a vitamin D_3 decomposition product bactericidal against *Helicobacter pylori*

Kouichi Hosoda, Hirofumi Shimomura, Kiyofumi Wanibuchi, Hisashi Masui, Avarzed Amgalanbaatar, Shunji Hayashi, Takashi Takahashi and Yoshikazu Hirai

Supplementary Information

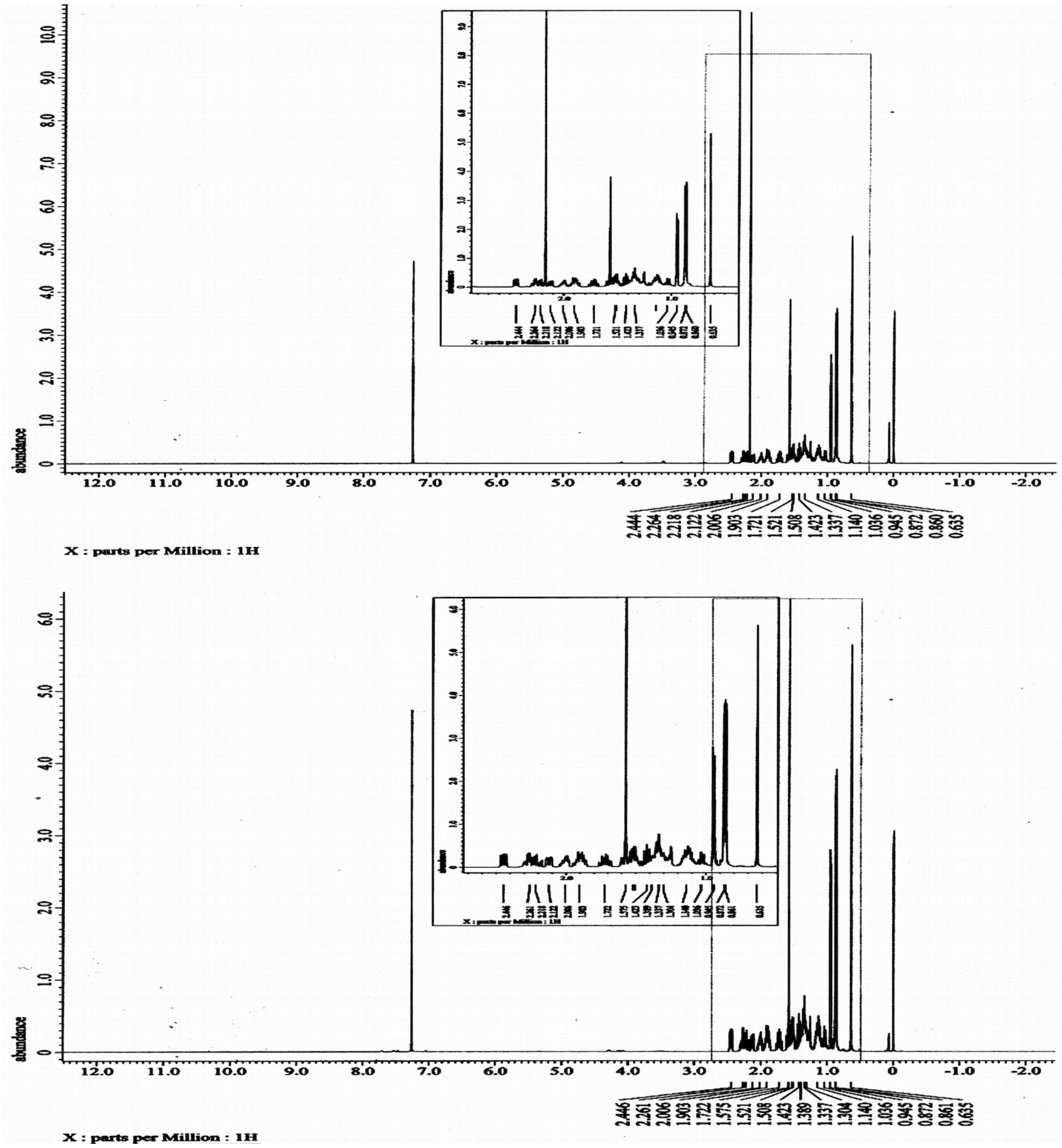

Fig. S1. Comparison of ^{1}H NMR signals of the purified VDP1 and synthesized VDP1

Upper panel denotes the signals of chemical shifts detected in ^{1}H NMR analysis of the purified VDP1. Lower panel denotes the signals of chemical shifts detected in ^{1}H NMR analysis of the synthesized VDP1.

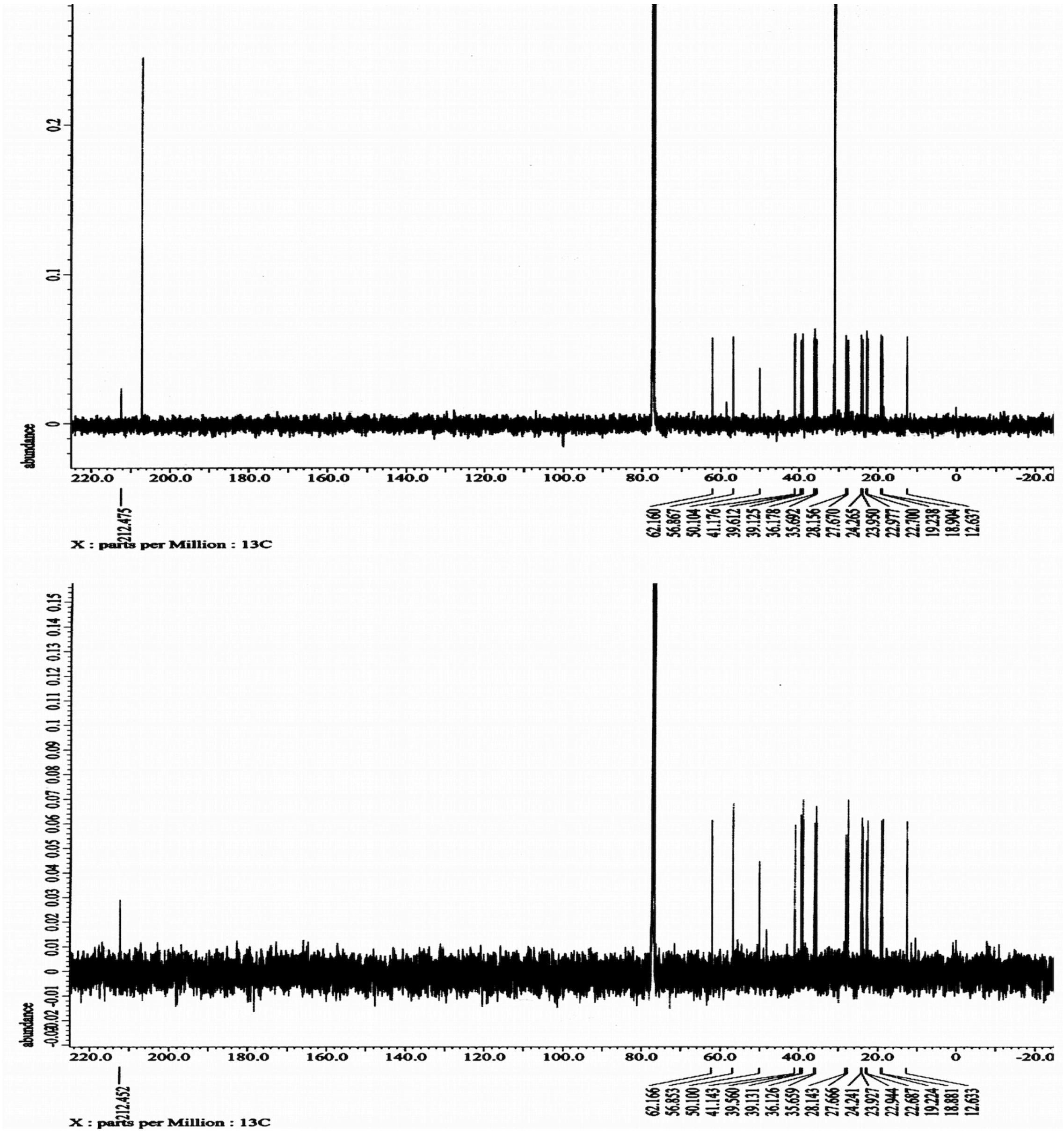

Fig. S2. Comparison of ^{13}C NMR signals of the purified VDP1 and synthesized VDP1

Upper panel denotes the signals of chemical shifts detected in ^{13}C NMR analysis of the purified VDP1. Lower panel denotes the signals of chemical shifts detected in ^{13}C NMR analysis of the synthesized VDP1.

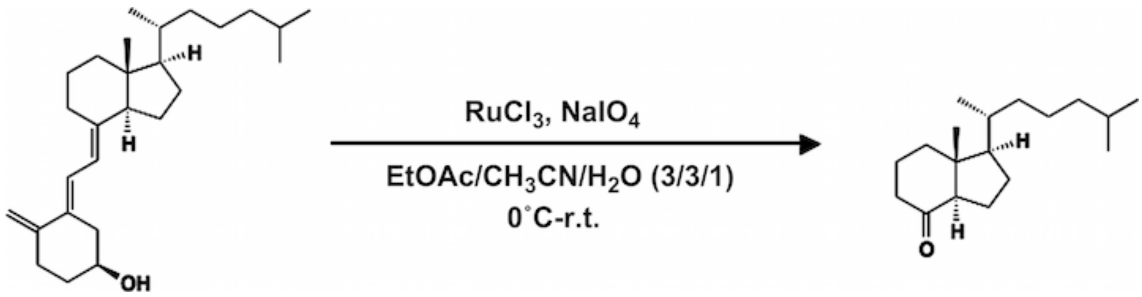

Fig. S3. Synthesis of VDP1 from vitamin D_3 via the catalyzed oxidation reaction

Ruthenium chloride ($RuCl_3$) was used as a catalyst. Sodium periodate ($NaIO_4$) was used as an oxidizer.

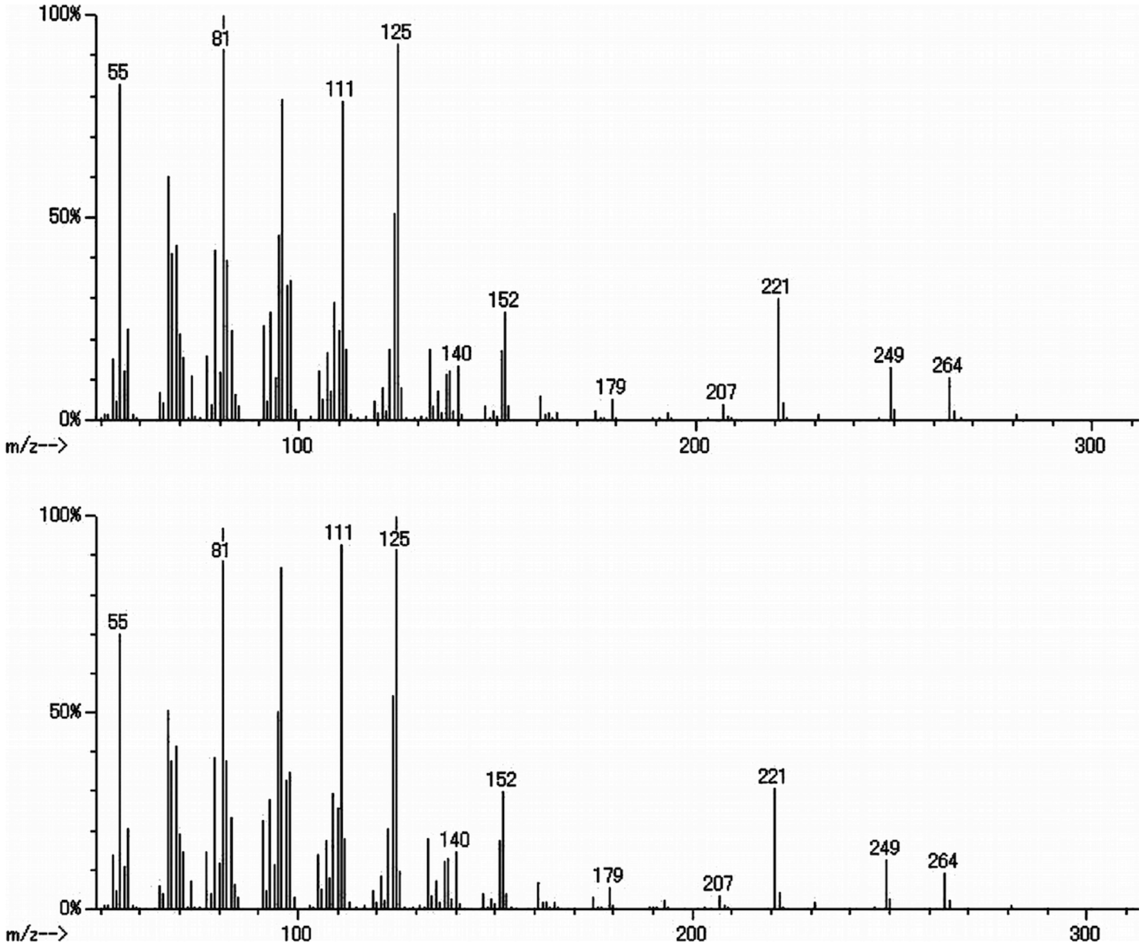

Fig. S4. Comparison of GC-MS spectra of the purified VDP1 and synthesized VDP1

Upper panel denotes the mass spectra in GC-MS analysis of the purified VDP1. Lower panel denotes the mass spectra in GC-MS analysis of the synthesized VDP1.
